# Comment on “Vertebral Arteriovenous Fistula: An Unwelcome Thrill”

**DOI:** 10.1155/2018/4509435

**Published:** 2018-07-19

**Authors:** Stephen M. Foreman, Michael J. Stahl

**Affiliations:** Private Practice of Chiropractic, USA

The publication titled “Vertebral Arteriovenous Fistula: An Unwelcome Thrill” by Edwards and his colleagues provided a review of the etiology, pathophysiology, and management of vertebral AV fistulae [1].

Our primary concern centers on the discussion of the possible etiology of their patient's vertebral AV fistulae and its possible relationship to chiropractic manipulation. Edwards et al. claim that “numerous prior cases of vertebral AVFs report induction by chiropractic manipulation” which is the genesis of our communication. The patient in Edwards et al. case report had sought chiropractic care for chronic neck pain. Although their patient “did not associate onset of her symptoms with any known physical trauma” Edwards et al. stated that “it is therefore plausible that our patient's fistula was caused by unreported chiropractic manipulation, although this patient's fistula occurred in the distal cervical portion of her vertebral artery, where previous studies have determined that spontaneous vertebral AVFs form most commonly.”

We believe Edwards et al. incorrectly cited our case report [2] as scientific evidence the literature contains “numerous prior cases of vertebral AVFs report induction by chiropractic manipulation.” Our case report made no such connection with spinal manipulation. In fact, we argued the* opposite*. We observed that the patient in our case report suffered from an intracranial AVF, an anatomical location immune to any effects of spinal manipulation. Our case report also observed that “attending physicians initially believed the patient had experienced a rare complication from cervical manipulation, an impression that prevailed for several days, delaying proper intervention. The location and breadth of the patient's ischemia and the absence of similar cases in the literature, should have argued against chiropractic manipulation as the cause of the patient's ascending myelopathy. The admitting physician's prolonged focus on a possible complication from chiropractic manipulation may be characterized as an anchoring bias.”

In conclusion, we again thank Edwards et al. for providing an excellent overview of an uncommon vascular disorder. Edwards et al. held the role of an unreported chiropractic manipulation as “plausible” for the AVF in their case report. Contrary to the assertion of Edwards et al., our case report does not make an association between chiropractic manipulation and vertebral AVFs. Our case report demonstrated that the potential for attending clinicians to formulate inaccurate etiological opinions of causation is based solely on temporal association in patients with a history of receiving chiropractic manipulation.

## References

[1] Edwards M. K., Christenson E. N., Corliss B. M., Polifka A. J., Allen B. R. (2017). Vertebral arteriovenous fistula: an unwelcome thrill. *Case Reports in Emergency Medicine*.

[2] Foreman S. M., Stahl M. J., Schultz G. D. (2013). Paraplegia in a chiropractic patient secondary to atraumatic dural arteriovenous fistula with perimedullary hypertension: case report. *Chiropractic & Manual Therapies*.

